# Revolutionizing cleft lip and palate management through artificial intelligence: a scoping review

**DOI:** 10.1007/s10006-025-01371-1

**Published:** 2025-04-10

**Authors:** Cristhian David Barreto Zambrano, Mariana Arias Jiménez, Angela Gabriela Muñoz Rodríguez, Erwin Hernando Hernández Rincón

**Affiliations:** 1 School of Medicine, Universidad de La Sabana, Chía, Cundinamarca Colombia; 2 Department of Family Medicine and Public Health, School of Medicine, Universidad de La Sabana, Campus Universitario Puente del Común, Km 7 North Highway of Bogotá,, Chía, Cundinamarca Colombia

**Keywords:** Artificial intelligence, Expert systems, Machine learning, Cleft palate, Cleft lip

## Abstract

**Purpose:**

Not much is known about the applications of artificial intelligence (AI) in cleft lip and/or palate. We aim to perform a scoping review to synthesize the literature in the last 10 years on integrating AI in the approach to this condition and highlight aspects of research into its prediction, diagnosis and treatment.

**Methods:**

A search was performed via PubMed, Science Direct, Scopus, and LILACS from 2014 to 2024, in which 649 articles were identified, and 3 studies were identified via the snowball method; the title and abstract were identified, and 35 articles were obtained for full reading. Finally, 25 studies were selected after applying the inclusion and exclusion criteria to execute this review.

**Results:**

The articles reviewed included different types of studies, with observational and experimental studies being frequent and systematic reviews and narratives being less frequent. Similarly, there was evidence of a generalized distribution, with a greater concentration in the United States. These studies were analyzed according to the use of AI applied to cleft lip/palate, obtaining 6 subcategories, including diagnosis, prediction, treatment, and education, in which different types of AI models were included, most frequently using deep learning and machine learning.

**Conclusion:**

These technologies promise to optimize the care of patients with this condition. Although current advances are promising, further research is essential to expand and refine their beneficial use. AI has driven significant advances in various stages of the cleft lip and/or palate approach, integrating tools such as assisted algorithms, genetics-based predictive models, and advanced surgical planning.

## Introduction

AI has been one of the essential technological advances of recent decades [1]. It acquires knowledge by analyzing large volumes of data to apply to different fields, allowing it to solve problems [2]. AI is described as a network of processors that seek to imitate biological systems, in which mathematical models applied to the data as a whole are used to make human decisions [3]. The use of this tool in the medical field has increased since it allows the processing of large amounts of information and identifies previously unknown data, which facilitates diagnosis, image interpretation, therapeutic approaches, and the prediction of medical outcomes [4].

According to the National Institute of Health, Cleft Lip and/or Palate (CL/P) is one of the most common orofacial malformations, with an estimated global prevalence of approximately 4.6 million [3, 5]. A cleft lip (CL) is defined as a fissure of variable size that extends through the lip, alveolus, and nasal floor, secondary to a disturbance in the fusion of the maxillary-frontonasal processes. Additionally, a Cleft Palate (CP) is defined as a defect in the fusion of the palatine processes of the maxillary bone. These alterations can present jointly or in isolation; CP is greater in women than in men, but CL/P is 2 times greater in men than in women [6]. CP or CL may present unilaterally or bilaterally and may or may not be associated with different syndromes [7].

Subcategories are mentioned for reporting incidence, where 1 in every 1,600 children will have CL/P, 1 in every 2,800 will be born with CL without CP and 1 in every 1,700 will present only with CP, highlighting a higher incidence in underdeveloped countries; however, Sub-Saharan Africa, Middle East/North Africa, and South Asia stand out for their high incidences [3, 5, 8]. Additionally, in rural areas, access to treatment tends to be delayed until older individuals are reached [9].

This condition contributes significantly to worldwide oral health morbidity since it not only is an anatomical and aesthetic defect but can also generate functional problems at the level of language and hearing. This leads to long-term deterioration of health and therefore in the quality of life of these patients [3], in addition to implications at the psychological, family, and social levels [10].

Although CL/P is the most common craniofacial anomaly in newborns, a clear etiology of this congenital defect is unknown, and it has been recognized that multiple factors are involved, whether genetic, environmental, or a combination of both [11]. For this reason, recommendations regarding prevention are variable; however, early interventions clearly result in a lower rate of complications [12], and the application of AI could represent a way to optimize different processes to obtain better outcomes under these conditions [13]. Nevertheless, since the use of this technology in this field is still novel, information remains limited. Therefore, it was considered pertinent to conduct a type of study that allows mapping the available evidence as a scoping review, with the aim of synthesizing the past decade’s literature on AI integration in CL/P care, identifying new uses on prediction, diagnosis and treatment, for improved outcomes applied to clinical practice, and recognizing gaps and potential opportunities for future research.

## Methods

**Fig. 1:**
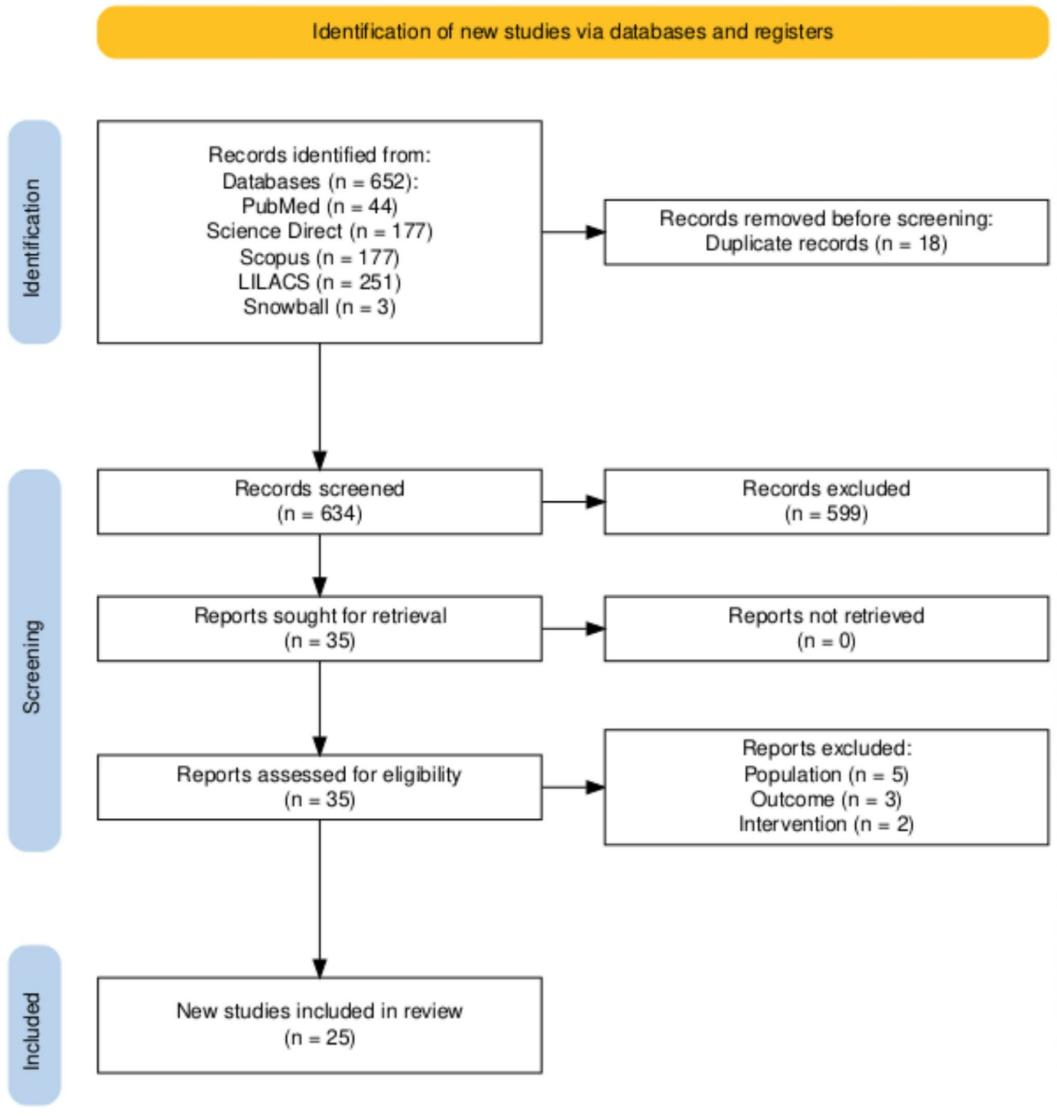
PRISMA flow diagram for the study selection process. Created using Shiny app [17, 18]

A scoping review was conducted in December 2024, with the aim of answering the following research question: what applications have been made of artificial intelligence in addressing cleft lip and/or palate?, mapping the literature, and identifying opportunities for future research. The protocol was registered in The Open Science Framework (OSF) [14] on February 17, 2025, under the registration number: osf.io/e324h. Additionally, to ensure the reproducibility and rigor of this research, the methodological approach proposed by Arksey and O’Malley [15] supplemented by the Joanna Briggs Institute (JBI) [16] guidelines for scoping reviews was followed. In addition, the Preferred Reporting Items for Systematic Reviews and Meta-Analyses extension for Scoping Reviews (PRISMA-ScR) [17, 18] was used as a guide for reporting the search process and findings (Fig. 1). The steps for the development of the present study are described below.

### Identifying the search question and relevant studies

Using Medical Subject Headings (MeSH) and Health Science Descriptors (DeCS) terms such as “Artificial Intelligence”, “Cleft Lip” and “Cleft Palate”, in combination with AI synonyms not recognized by the Virtual Health Library (VHL) and Boolean operators, a literature search was performed in databases such as PubMed, Science Direct, Scopus and LILACS, selected for their broad coverage of biomedical literature as well as for their coverage of scientific articles in different disciplines, which allowed a broad and reliable exploration of the literature. Table 1 shows the search strategies and filters applied in each database to ensure the reproducibility of the process. Articles published between the years 2014 and 2014 and written in English or Spanish were included. Given the small number of publications identified through the search described above, snowball methodology was implemented to broaden the search, identifying references of the selected articles that could contain useful information to solve the research question.

**Table 1:** Search methods for each database. Own elaboration

Database	Search strategy	Filters
PubMed	ALL FIELDS ([Artificial Intelligence] OR [Cognitive Computing] OR [Intelligent Systems]) AND ([Cleft Palate] OR [Cleft Lip)]	Publication date: 2014–2024 Full-text availability Language: Spanish and English
Science Direct	([Artificial Intelligence] OR [Cognitive Computing] OR [Intelligent Systems]) AND ([Cleft Palate] OR [Cleft Lip)]	Publication date: 2014–2024 Full-text availability Document type: Article and Review
Scopus	ALL FIELDS ([Artificial Intelligence] OR [Cognitive Computing] OR [Intelligent Systems]) AND ([Cleft Palate] OR [Cleft Lip)]	Publication date: 2014–2024 Full-text availability
LILACS	TITLE, RESUME, ABSTRACT (Artificial Intelligence) OR (Cognitive Computing) OR (Intelligent Systems) AND (Cleft Palate) OR (Cleft Lip)	Publication date: 2014–2024 Full-text availability Language: Spanish and English

### Selection criteria

The selection criteria for the articles were established following the population, concept, and context (PCC) strategy recommended by the Joanna Briggs Institute [16]. These criteria cover patients eligible for screening, diagnosis, and treatment of CL/P. However, patients requiring additional interventions for primary closure, such as orthodontic procedures or orthognathic surgery, are excluded, since these secondary procedures belong to specialties with highly technical and specific approaches, thus making it difficult to interpret and generalize the findings. Additionally, variability in secondary treatment protocols could lead to inconsistencies in the results, complicating the evaluation of the impact of artificial intelligence in the initial phase of treatment.

In addition, all the artificial intelligence techniques used, whether neural networks, machine learning, deep learning, or natural language processing, were included to obtain as much information as possible on the use of this technology in the field of interest. In addition, various types of studies, including observational, experimental, and reviews, were considered to provide a comprehensive synthesis of the topic and to extract relevant evidence on the use of AI in CL/P. The selection criteria are highlighted in detail in Table 2.

**Table 2:** Selection criteria. Own elaboration

	Inclusion criteria	Exclusion criteria
Population	Individuals susceptible to screening, diagnosis, and treatment of cleft lip and/or palate.	Individuals requiring additional surgical management other than primary closure.
Concept	All artificial intelligence (AI) techniques, such as machine learning, deep learning, neural networks, and natural language processing. Use of AI in the context of cleft lip and/or palate.	Articles that only discuss surgical management in patients with cleft lip and palate that have already been treated Articles that do not discuss AI in the context of cleft lip/palate.
Context	Cleft Lip and/or Palate	Other congenital craniofacial anomalies.
Type of Study	Clinical trials, systematic reviews, scoping reviews, cross-sectional descriptive or interventional studies, and narrative reviews.	Protocol studies, conferences, letters to the editor, and case reports. Studies without access to full text.

### Study selection

After following the previously described methodology and importing the articles obtained from the search into RAYYAN, a tool designed to streamline article filtering in literature reviews [19], the study selection process was carried out in three steps:

(1) An initial identification was performed, considering only the titles and abstracts of the studies to identify potentially relevant studies, followed by removing duplicates. (2) Next, screening, which was independently conducted by two investigators who worked independently and blindly, was performed based on the previously established inclusion and exclusion criteria. Discrepancies arising in this first stage were resolved by a third investigator, who intervened to determine the inclusion or exclusion of the studies in conflict. (3) Finally, the selected articles were thoroughly reviewed for final evaluation, considering their specificity and relevance to the objectives of this research. This rigorous process ensured the quality and consistency of the chosen studies, confirming their relevance to the proposed scoping review.

### Compiling, summarizing, and presenting results

After the articles that had been fully reviewed were selected, data extraction was carried out by creating a table to organize the information. This table includes key data from each selected study, such as bibliographic details (author, year, country) to determine the temporal evolution of available research and its geographical distribution to identify trends in the use of artificial intelligence, methodological design, type of artificial intelligence used, and main findings relevant to addressing the objective of this scoping review (Table 3).

**Table 3:** Applications of artificial intelligence in cleft lip and/or palate. Own elaboration

Títle	Author	Year	Country	Type of Study	AI-technique	Document Summary
**Artificial Intelligence in the General Approach to Cleft Lip and/or Palate.**						
Current Applications of Artificial Intelligence in Cleft Care: A Scoping Review	Dhillon et al. [3]	2021	Contributions from India, Taiwan and Saudi Arabia	Scoping Review	Machine Learning	Identifies clefts and their morphologic features, development of sequential plaques, and identification of markers for surgical management, as well as speech evaluation in individuals with cleft lip and palate using AI.
Harnessing the Power of Artificial Intelligence in Cleft Lip and Palate: An In-Depth Analysis from Diagnosis to Treatment, a Comprehensive Review	Almoammar et al. [13]	2024	Saudi Arabia	Comprehensive Review	Deep Learning	Describes the findings in the literature on the use of AI, especially Deep Learning models, in fields such as diagnosis, treatment, and prediction, finding that the application of this technology revolutionizes and improves the approach to this condition.
Clinical Applications of Artificial Intelligence and Machine Learning in Children with Cleft Lip and Palate: A Systematic Review	Huqh et al. [21]	2022	Malaysia	Systematic Review	Machine Learning	To analyze the different applications of Artificial Intelligence and Machine Learning models to evaluate their diagnostic performance in children with cleft lip and palate.
**Artificial Intelligence in the Diagnosis of Cleft Lip and/or Palate.**						
Deep-learning systems for diagnosing cleft palate on panoramic radiographs in patients with cleft alveolus	Kuwada et al. [22]	2022	Japan	Experimental Study	Deep Learning	Creation of Deep Learning-based models using Detect Net and VGG-16 to diagnose cleft palate in cleft alveolus patients using panoramic radiographs.
The utilization of artificial intelligence in enhancing 3D/4D ultrasound analysis of fetal facial profiles	Bachnas et al. [23]	2024	Indonesia, Croatia	Narrative Review	Deep Learning	Performs a narrative review of the integration of artificial intelligence in 3D/4D ultrasound analysis of fetal facial profiles, including cleft lip and palate, leveraging Machine Learning and Deep Learning algorithms, to improve performance in medical approach.
Evolving the Era of 5D Ultrasound? A systematic literature review on the applications for artificial intelligence ultrasound imaging in obstetrics and gynecology	Jost et al. [24]	2023	Germany	Systematic Review	Deep Learning	A systematic review of the literature was performed evaluating the use of ultrasound associated with the use of artificial intelligence in the context of obstetrics and gynecology, mentioning that the inclusion of artificial intelligence reduces access and training limitations and proposes the analysis of cleft lip and palate ultrasound associated with AI.
Recognition of fetal facial ultrasound standard plane based on Texture Feature Fusion	Wang et al. [25]	2021	China, United States	Observational Study	Machine Learning	In this study, a texture feature fusion method (LH-SVM] is proposed for the automatic recognition and classification of FFUSP. This study was considered taking into account the ultrasonographic diagnostic process for ultrasonographic facial identification to reduce the gaps in the manual method performed by doctors.
**Artificial Intelligence in Cleft Lip and/or Palate Prediction**						
Genetic Risk Assessment of Nonsyndromic Cleft Lip with or without Cleft Palate by Linking Genetic Networks and Deep Learning Models	Kang et al. [26]	2023	South Korea	Observational Study	Deep Learning	Use of Deep Learning models when integrating genetic networks (GANNE] for genetic risk prediction in non-syndromic cleft lip with or without cleft palate.
The Contemporary Management of Cleft Lip and Palate and the Role of Artificial Intelligence: A Review	Marya et al. [45]	2022	Contributions from Cambodia, Malaysia, India, Armenia, Thailand	Narrative Review	Deep Learning	They develop a review, showing different studies in which it has been shown how artificial intelligence can simplify different stages of cleft lip and palate management. It highlights the use of predictive models for facial clefts in the prenatal period and how these have high levels of accuracy.
Machine Learning Models for Genetic Risk Assessment of Infants with Non-Syndromic Orofacial Cleft	Zhang et al. [27]	2018	China	Observational Study	Machine Learning	Development of Machine Learning systems to assess the genetic risk of non-syndromic orofacial clefting in infants using single nucleotide polymorphisms.
DeepFace: Deep-learning-based framework to contextualize orofacial-cleft-related variants during human embryonic craniofacial development	Dai et al. [29]	2024	United States	Experimental Study	Deep Learning	Development of software based on deep neural networks to analyze genetic variants related to orofacial clefts. It uses data from the genetics of craniofacial development to predict the functional impact of variants, highlighting those that are related to and characteristic of these craniofacial malformations.
Machine learning in the prediction of genetic risk of nonsyndromic oral clefts in the Brazilian population	Machado et al. [46]	2020	Brazil	Observational study	Machine Learning	Use of Machine Learning to detect possible interactions between 13 single nucleotide polymorphisms as predictors of genetic risk of non-syndromic cleft lip with or without cleft palate in Brazilian population.
Cleft prediction before birth using deep neural network	Shafi et al. [51]	2020	Pakistan, Saudi Arabia	Experimental study	Deep Learning	Creation and application of a questionnaire in conjunction with a Machine Learning-based model for the prediction of orofacial clefts in the prenatal period in Pakistan. The model achieved an accuracy of 92.6%. Relevant risk factors and their respective recommendations are also highlighted.
Gene-Gene interaction among WNT genes for an oral cleft in trios	Li et al. [28]	2015	United States and Thailand	Observational Study	Machine Learning	Analysis of 18 genes of the WNT signaling pathway to identify significant genetic interactions that could influence the risk of cleft lip/palate by Machine learning.
**Artificial Intelligence in the evaluation of Aesthetic Sequelae in patients with Cleft Lip and/or Palate.**						
Facial attractiveness of cleft patients: a direct comparison between artificial-intelligence-based scoring and conventional rater groups.	Patcas et al. [30]	2019	Switzerland, China	Observational Study	Deep Learning	Assessment of facial attractiveness employing neural network algorithms in patients with cleft lip and palate.
Where is the artificial intelligence applied in dentistry? Systematic review and literature analysis	Thurzo et al. [31]	2022	Slovakia, Czech Republic	Systematic Review	Machine Learning	A systematic review was carried out with two main objectives: First, to evaluate how often artificial intelligence was used in the context of dental literature and to evaluate in these different publications by topic, it became evident that its main use is on esthetic and functional results.
**Artificial Intelligence in the Treatment of Cleft Lip and/or Palate.**						
Interpretable artificial intelligence for classification of alveolar bone defect in patients with cleft lip and palate	Miranda et al. [32]	2023	Contributions from Brazil, Italy, Saudi Arabia, and the United States	Experimental study	Machine Learning	Evaluation of the Fly-by-CNN algorithm analyzing 3D objects and 2D images and sending them to a convolutional neural network to analyze the severity of alveolar bone defects in patients with cleft lip and palate
Machine Learning Demonstrates High Accuracy for Disease Diagnosis and Prognosis in Plastic Surgery	Mantelakis Et al. [33]	2021	United Kingdom	Systematic Review	Machine Learning	Analysis of the applications of Machine Learning models in the prediction of surgical management of pathologies in plastic surgery including cleft palate.
Harnessing the Power of Artificial Intelligence to Teach Cleft Lip Surgery	Sayadi et al. [34]	2022	United States	Experimental study	Deep Learning	AI algorithm analysis to detect anthropometric landmarks and anatomical landmarks to understand the anatomy of the nasolabial cleft and design various types of nasolabial surgical repair in cleft lip patients.
Artificial intelligence applications and ethical challenges in oral and maxilla-facial cosmetic surgery: A Narrative review	Rokhshad et al. [36]	2023	Contributions from Germany, United States, South Korea, Iran	Systematic Review	Machine Learning	A narrative review was conducted on the applications and ethical challenges in the context of the use of artificial intelligence for the development of cosmetic maxillofacial and oral surgery, in order to provide the fundamental technical elements to surgeons to understand the potential of using this tool.
Smartphone-based scans of palate models of newborns with cleft lip and palate: Outlooks for three-dimensional image capturing and machine learning plate tool	Santos et al. [37]	2024	Brazil, Switzerland	Experimental study	Machine Learning	This study evaluated the performance of smartphone scanning applications in acquiring 3D impressions of cleft palate models and a Machine Learning tool was validated to compute pre-surgical plates in an automated fashion
Personalized quantification of facial normality a machine learning approach	Boyaci et al. [35]	2020	Qatar	Experimental study	Deep Learning	A computerized model was created to produce a realistic image of any facial image presented, which objectively focuses the treatment on the patient’s own facial normality and features in order to provide the surgeon with better planning and patient education tools.
Detection and classification of unilateral cleft alveolus with and without cleft palate on panoramic radiographs using a deep learning system	Kuwada et al. [47]	2021	Japan	Experimental study	Deep Learning	Development of two Deep Learning models for the diagnosis and classification through panoramic radiographs of cleft alveolus in patients with and without cleft palate. It was found that these models, although they have limitations, represent a potential aid for human observers in the approach and therapeutic planning of cleft alveolus.
**Artificial Intelligence in Cleft Lip and/or Cleft Palate Education.**						
Easing the Burden on Caregivers- Applications of Artificial Intelligence for Physicians and Caregivers of Children with Cleft Lip and Palate	Chaker et al. [38]	2024	United States	Observational Study	AI Language Models (Chat GPT)	Evaluation of Chat GPT’s ability to produce responses, educational and support materials to parents and caregivers of cleft lip and palate patients to decrease the emotional burden.
Global birth defects app: An innovative tool for describing and coding congenital anomalies at birth in low-resource settings	Dolk et al. [39]	2021	Contributions from the United Kingdom, Croatia, Uganda, the United States, Denmark, Colombia, Brazil, India, Switzerland	Observational Study	AI Language Models	Development of applications on Android and Apple devices for surveillance, prevention, and care of congenital anomalies through AI and the implementation of other AI domains for possible diagnosis of genetic syndromes.

The articles included in this table were independently evaluated by two researchers to minimize errors and biases, with a third researcher responsible for resolving any discrepancies. Finally, we used Mendeley [20], a reference management tool that allows storing, organizing, annotating, sharing, and citing the extracted data. The selected studies met the preestablished inclusion criteria, prioritizing full-text articles that provided sufficient data to answer the research question, ensuring a comprehensive analysis of the reported information.

## Results

The initial search identified 652 articles, with 18 duplicates removed, leaving 634 for the initial review. A screening process excluded 599 articles on the basis of title and abstract examination, leaving 35 articles with full access for detailed evaluation according to the inclusion and exclusion criteria. Of these, 10 were excluded because of population (*n* = 5), intervention (*n* = 2), or outcome (*n* = 3) criteria. Ultimately, 25 articles were included for discussion.

The most frequent types of studies were observational and experimental (*n* = 8, 32% each), followed by systematic reviews (*n* = 5, 20%). Other types of studies included narrative reviews (*n* = 2, 8%), scoping reviews and narrative reviews (*n* = 1, 4% each). The geographic distribution of publications revealed no specific regional concentration but rather a generalized pattern. The leading contributors were the United States (*n* = 7, 25.93%), Brazil (*n* = 4, 14.81%), and Saudi Arabia (*n* = 4, 14.81%), followed by India, Switzerland, and China, each contributing (*n* = 3, 11.11%). In addition, there has been a notable increase in studies in this field over the last four years, accounting for 76% of the total (*n* = 19).

Similarly, regarding the use of AI, deep learning (DL) was the most commonly used model, including a subtype called deep neural networks (NNs) (*n* = 12, 44%), as well as machine learning (ML). AI language models are also used, with Chat GPT allowing the creation of answers and educational and support materials (*n* = 1, 3%). The use of AI without additional specifications was described in two of the articles (*n* = 2, 7%).

The articles were classified into 6 subcategories: *“General approach*,* diagnosis*,* prediction*,* evaluation of aesthetic sequelae*,* treatment and CL/P education”*, as described in Table 3. First, the general approach to these craniofacial anomalies is discussed, with emphasis on AI and its different applications in CL/P, including diagnostic performance, where it has been shown that the use of computer-aided diagnosis [2] and convolutional NNs could emulate medical visual inspection and automate and refine diagnostic processes [13, 21].

Concerning the diagnosis of CL/P, different models based on AI that allow a diagnosis through panoramic radiographs [22] and 3D ultrasound analysis [23] describe the use of 5D ultrasound [24] and propose the texture feature fusion method [25] to evaluate which is the best method to define this diagnosis.

Multiple AI models have been used to predict this pathology, integrating genetic networks [26], evaluating single-nucleotide polymorphisms, different WNT signaling pathways [27, 28], and software based on NNs [26]. These models identify genes associated with this entity, allowing the prediction of the functional impact of orofacial clefts [29]. Similarly, genes that contribute to the metabolism of different micronutrients have been described, which, when deficient, represent a risk factor for orofacial clefts [27].

Additionally, the articles were classified based on the evaluation of AI in aesthetic sequelae in patients with CL/P and focused on facial asymmetry and scar management after surgery, which impacts psychosocial well-being [30]. Intraoral scanning and prosthesis placement significantly improved aesthetic and functional issues [31]. Treatment algorithms analyzing images and dental characteristics facilitate the evaluation of severity, analysis of anthropometric marks, and surgical planning by quantifying and classifying defects [32–35], along with exploring applications, ethical challenges, and new uses [36–37].

Finally, the field of education in CL/P was included, where AI made it possible to evaluate responses by the ChatGPT to educate caregivers about this pathology [38]. The development of new digital tools in mobile devices was also observed to educate health personnel on congenital anomalies via AI while contributing to the surveillance, prevention, and timely care of these pathologies [39].

## Discussion

Given the advancements and improvements achieved by incorporating AI into CL/P, in addition to the rise of the literature in the field, the previously outlined results categories will now be discussed:

### Artificial intelligence in the general approach to cleft lip and/or palate

Considering the genetic aspect, Huqh et al. [21] and Dhillon et al. [3] mentioned observational studies approaching this pathology from a genetic perspective, obtaining findings such as MTHFR and RBP4 mutations discovered via ML algorithms, as well as the presence of single nucleotide polymorphisms (SNPs) that significantly predict the risk of this condition [27.

Additionally, Dhillon et al. [3] mentioned how expositional factors could vary depending on geographic location and ethnicity, and another study that used AI sought to predict their occurrence according to family history, providing significant results. In contrast, Almoammar et al. [13] reported significant gene‒gene interactions (WNTs) with DL and reiterated the importance of considering gene‒based interactions as triggering factors of the pathology to be treated.

On the other hand, as mentioned by Huqh et al. [21], one of the concerns surrounding CL/P is the discrepancy in maxillary growth. In relation to this, he noted that comparative studies on cephalometric calculations through AI-based software have been proposed, which ultimately has an impact on better surgical planning. They also mention AI-based applications for the classification of hypernasality, another common clinical problem in CL/P patients.

In terms of diagnosis, Almoammar [13] highlighted the use of NNs in the collection of relevant data from diagnostic images, allowing early identification of CL/P, as well as a follow-up of its progression. As an example, he mentioned the implementation of trained convolutional NN models to detect landmarks in images and classify defect severity, achieving an accuracy of up to 89%. These optimal results in terms of image analysis capacity are consistent with other reviews on AI applications in the diagnosis of congenital anomalies, such as the one performed by Yousefpour Shahrivar et al. [40], which mentioned the use of NN models for the analysis of echocardiograms for the detection of congenital heart disease, which reached 85% accuracy, indicating that this AI model has great potential in the diagnostic field.

### Artificial intelligence in the diagnosis of cleft lip and/or palate

As noted in the previous section, one of the topics that has attracted the most attention in recent times has been the use of convolutional NNs in diagnostic imaging; DL algorithms allow a therapeutic approach that, in some cases, can match or even surpass expert diagnoses as demonstrated by Kuwada et al. [25] in their experimental study where it was determined that DL models present a higher accuracy in detecting defects in panoramic radiographs, with an AUC > 0,9 compared to radiologists with an AUC ≤ 0,7. While AI may present better performance, reviews such as the one conducted by Gampal et al. [41] highlight how AI can complement the field of radiology rather than replace it, with utility such as removing artifacts from images or taking measurements on segmented images.

The use of AI in conjunction with 2D, 3D, and 4D ultrasonography enhances the ability to diagnose orofacial malformations by capturing details that may be missed by the human eye [24, 25]. In relation to the different sonographic dimensions, Jost et al. [24] mentioned important advances in the automation of identification in standard planes in 2D ultrasonography to diagnose CL/P. Moreover, Bachnas et al. [23] emphasized that the use of AI with human feedback in the loop in conjunction with 4D ultrasonography allows the incorporation of automation into the diagnostic process.

Similarly, Wang et al. [25] proposed a model for feature extraction from such images to perform predictive classification via a support vector machine (SVM), a technique derived from ML, with an accuracy of up to 94.67%, which highlights how these automatic detection methods can greatly contribute to the ultrasonographic diagnosis of CL/P. However, the implementation of this technology in the diagnosis of malformations still faces some limitations, as noted by Ravelo et al. [42], such as the heterogeneity of the analyzed images due to the ethnic differences in the analyzed populations, which hinders the generalization of the results, as well as the lack of studies that analyze images that are not only two-dimensional, since cephalometric measurements in 3 dimensions would provide more information.

Therefore, AI in the field of detection of this type of pathology has many uses, as evidenced in the review by Fonseca et al., who highlighted the use of different kinds of artificial intelligence under different modalities, such as deep learning or machine learning, for the diagnosis of multiple craniofacial anomalies [43]. This coincides with the results obtained in this review, where the studies opt for deep learning applied to images for the optimization and automation of CL/P diagnosis [23].

For example, AI, particularly convolutional neural networks, has also been applied to craniosynostosis and has proven highly useful for classifying its different subtypes via 2D images [44]. As with CL/P, the use of these technological innovations is relatively recent, requiring larger datasets for proper AI training and broader generalization of its application.

### Artificial intelligence in the prediction of cleft lip and/or palate

In approximately 2010, hypotheses were developed via AI, in which genes linked to orofacial clefts were identified, and genome-wide association studies (GWASs) and SNP markers were used to predict genetic risk [26]. The polygenic risk score (PRS) sums alleles to predict disease risk [27]. ML algorithms facilitate risk prediction by detecting patterns and interactions in model inputs [26]. Kang et al. [26] evaluated the prognosis of models that were developed via DL techniques, including genetic algorithm-optimized neural networks (GANNE), to analyze the predictive power of this entity. The main SNPs were identified in the genetic association analysis; these data were used as input for DL, and GANNE allowed it to significantly improve the predictive performance.

A study evaluated 143 Korean patients with nonsyndromic cleft lip/palate (NSCL/P) and analyzed 92 SNPs via the GANNE method, along with other risk classification methods (PRS, random forest (RF), SVM, extreme gradient boosting, and NN), and the results revealed that GANNE provided the highest predictive power, which was 23% greater than that of PRS and 17% greater than that of NN. The key genes for NSCL/P genetic prediction included IRG 6, RUNX2, MTHFR, PVRL1, TGFB3, and TBX23 [45].

Similarly, a systematic review and meta-analysis in the Brazilian population evaluated genetic variants and revealed that BMP4 has a protective effect. Machado et al. [46] genotyped 72 SNPs and applied ML techniques, including RF and neural networks. The RF model identified 13 significant SNPs, achieving 94.5% accuracy in distinguishing NSCL/P patients, making it highly predictive of this condition in Brazil.

In the same way, Li et al. [28] studied risk loci for NSCL/P, focusing on 12 known loci. Using ML methods, including RF and logistic regression, they identified 25 top SNPs in Asians and 32 in Europeans with significant scores. WNT5B and MAFB were the top-ranked SNPs showing strong interactions in both populations.

Different diagnostic techniques using AI have been tested to detect this condition, taking into account risk factors described in the literature, such as family history, number of children, smoking habits, and history of spontaneous abortions, thus finding more than 90% accuracy in predicting fissures [45]. The proliferative expansion and complex morphogenetic events that contribute to facial development are very sensitive to environmental influences in conjunction with defective genetics. Additionally, several nutrients, such as folic acid and vitamin A, are known to prevent this condition [27].

Zhang et al. [27] studied 43 SNPs linked to NSCL/P and developed a genetic risk model via ML. They demonstrated how AI reduces biases from inconsistent odds ratios (ORs) across samples and accounts for homozygous and heterozygous risk alleles. The present study revealed that genes involved in folic acid and vitamin A metabolism, such as MTHFR and RBP4, had significant homozygous risk alleles in the control group. However, the high variability between the genetic prevalence of different populations limits the generalizability of these findings in CL/P prediction.

On the other hand, Dai et al. [29] evaluated human craniofacial epigenetic matrices via a DL model incorporating an NN specifically trained for cleft lip development. This deep facial model identifies associations between epigenetic features and long-range DNA sequence features, enabling the prediction of sequence alterations. The AI-based model provides quantitative measurements of skull-related SNPs during craniofacial development and reveals six high-risk craniofacial SNPs with a significant linear relationship between their epigenetic impact and the craniofacial development process.

GWAS for NSCL/P has identified multiple genes critical to the etiology of this congenital defect [32] Along with the various risk factors described, these findings help predict this craniofacial malformation, ensure proper attention, and contribute to prevention efforts [45].

### Artificial intelligence in the evaluation of aesthetic sequelae in patients with cleft lip and/or palate

Orofacial clefts require interdisciplinary management to ameliorate the negative side effects of the initial treatment and thus their impact on patient function and appearance. There is often scarring from surgical interventions and asymmetry around the nose and mouth, so there is a need to study the impact of evaluating facial attractiveness on the psychosocial well-being of patients and, therefore, to measure the outcome of the final treatment [30].

For patients with this condition, periodontal health is represented by stability, aesthetics, and teeth function. Thurzo et al. [31] discussed how AI manages to design automated search algorithms to analyze this type of periodontal disease and ultimately avoid aesthetic and functional problems, for example, how AI helps in the accurate matching of denture shade, as well as the optimization of dental implants.

### Artificial intelligence in the treatment of cleft lip and/or palate

Given the multiple domains involved in craniofacial anomalies, multidisciplinary management is essential, addressing issues such as language and hearing impairments, dentoalveolar alterations, and subsequent surgical approaches [32]. Diagnostic images are crucial for planning treatment for alveolar defects, but their interpretation can be challenging for medical staff. Therefore, AI has been evaluated for its potential to facilitate this task, improving the detection and classification of defects and consequently enhancing the accuracy and outcomes of surgical approaches.

An example of this is the study by Miranda et al. [32], in which the severity classification algorithm combined with convolutional neural networks was applied to CT images to identify key features that enhance classification accuracy. Similarly, Kuwada et al. [47], developed a computer-aided diagnostic model using DL techniques to detect cleft sockets on panoramic radiographs and classify them on the basis of the presence or absence of a concomitant cleft palate. Both studies highlight the potential of AI to streamline image analysis in medical practice.

However, previous studies acknowledge the limitation of a limited number of images and data poses, both for training programs, improving their performance, and for generalizing results [32, 47] Therefore, ensuring an adequate and diverse data sample is crucial when AI-based tools are used. A representative dataset not only improves their reliability in clinical settings but also enables broader applicability to the general population, helping to reduce healthcare disparities, as suggested by Khalifa et al. [48].

An important aspect of managing this congenital defect is determining which patients will require surgery and planning accordingly. Mantelakis et al. [33]. conducted a systematic review on ML in plastic surgery and reported that it can predict which cleft palate patients will need orthognathic surgery during preoperative planning. Additionally, they noted that surgical design, including anthropometric point marking, can vary on the basis of factors such as the surgeon’s experience. Sayadi et al. [34]. developed a deep learning model using convolutional neural networks to automate the placement of nasolabial markings to guide surgical design.

### Artificial intelligence in cleft lip and/or palate education

Another area that has benefited from AI in the CL/P context has been education, both to medical personnel and to patients and their families. Among caregivers of these patients, there can be distress and uncertainty about diagnosis, care, and treatment [38]. Chaker et al. [38] evaluated the potential of ChatGPT as a tool for family and caregiver education, finding that it can be a source of mostly accurate information that could contribute to reducing stress and emotional burden. This capability is also supported by studies such as the one conducted by Paran et al. [49]., where this AI was shown to outperform other methods in terms of quality, conciseness and accuracy in providing information for parents of patients with congenital anomalies compared with information sheets conducted by physicians.

In addition, Dolk et al. [39]. described the use of the “global birth defects” application, which allows health personnel to learn and guide the classification of congenital defects visible at birth, including CL/P, especially in areas with difficult access to specialists, allowing early diagnosis and timely management. However, the accuracy of the information generated by these tools should be monitored to avoid misinformation.

The studies highlighted the diversity of AI applications in the context of CL/P, which has allowed this tool to be used in diagnosis, treatment, esthetic evaluation and education, demonstrating adaptability. However, some of these applications remain in experimental phases and are not yet widely applied in clinical settings [3, 13]. Likewise, for many of these implementations, the data used to train AI models are still limited, affecting their performance and use [22, 25].

Correspondingly, there are limitations in terms of global applicability, as most of the results are from the United States, Brazil, and Saudi Arabia. This highlights the need to collect data in understudied regions, such as Latin America [50], where the integration of AI in the clinical setting faces several challenges. Among them, technological and socioeconomic barriers are identified, given that in many areas, difficulties persist in accessing advanced technologies and receiving training in AI [48]. The latter aspect is particularly relevant, as healthcare personnel lack specific training in the field, which represents a significant challenge, since effective implementation requires technical knowledge [34] that has not yet been fully incorporated into their preparation.

## Conclusions

The application of AI has advanced CL/P-related diagnosis, prediction, treatment, and education. Tools such as diagnostic algorithms, genetic variability modeling, and surgical planning help improve prevention and care. However, as this is an emerging topic, knowledge gaps exist, especially for data from underrepresented populations such as Latin Americans, and AI integration is limited by limited resources. Research should focus on more inclusive data collection and explore the development of accessible, low-cost technological solutions tailored to contexts with limitations.

Ethical issues also arise, particularly in relation to early prenatal diagnosis, which may lead to eugenic debates. Moreover, the potential dehumanization of medical care through AI highlights the need to question its role in decision making. Despite these challenges, AI shows promising advantages in terms of automation and research, offering significant benefits for cleft lip and/or palate care, so we propose that more detailed studies be conducted, such as systematic reviews or clinical trials in more specific areas, such as prenatal diagnosis, genetic risk assessment or optimization of secondary interventions such as orthognathic surgery, as research on these topics promises great benefits in the future for patients, clinicians and researchers alike.

## Data Availability

No datasets were generated or analysed during the current study.
